# Comparison of Thermal and Hydrotime Requirements for Seed Germination of Seven *Stipa* Species From Cool and Warm Habitats

**DOI:** 10.3389/fpls.2020.560714

**Published:** 2020-09-30

**Authors:** Rui Zhang, Kai Luo, Dali Chen, Jerry Baskin, Carol Baskin, Yanrong Wang, Xiaowen Hu

**Affiliations:** ^1^ College of Tropical Crops, Hainan University, Haikou, China; ^2^ State Key Laboratory of Grassland Agro-ecosystems, College of Pastoral Agriculture Science and Technology, Lanzhou University, Lanzhou, China; ^3^ Department of Biology, University of Kentucky, Lexington, KY, United States; ^4^ Department of Plant and Soil Sciences, University of Kentucky, Lexington, KY, United States

**Keywords:** seed germination, *Stipa*, temperature, water potential, habitat

## Abstract

Temperature and water potential are two important environmental factors influencing germination and subsequent seedling establishment. Seed germination requirements vary with species and with the environment in which the seeds are produced. *Stipa* species dominate large areas of the Eurasian zonal vegetation, but comparisons of germination requirements between *Stipa* species from different habitats is limited. We investigated the effects of temperature and water potential on seed germination of *S. grandis*, *S. purpurea*, and *S. penicillata* from habitats with low temperatures and relatively abundant rainfall (cool habitats) and *S. glareosa*, *S. breviflora*, *S. gobiea*, and *S. bungeana* from habitats with relatively high temperatures and low amount of rainfall (warm habitats). Seeds of species from cool habitats had a higher base (*T*_b_), optimal (*T*_o_), and maximum (*T*_c_) temperature than those of species from warm habitats, except for the base temperature of *S. purpurea*. Response of six tested *Stipa* species to water potential differed among species but not between habitats. Median water potential for germination was lowest for *S. bungeana*, *S. penicillata*, and *S. gobiea*. There was a negative correlation between hydrotime constant (*θ*_H_) and base water potential for 50% of the seeds of all species to germinate (*ψ*_b(50)_). Germination time of seven *Stipa* species in response to temperature and water was well predicted by thermal time and hydrotime models. Results of the present study on germination of these seven species of *Stipa* may provide useful suggestions for grassland restoration in different habitats.

## Introduction

Seed germination is the most critical stage in the life cycle of plants, and its correct timing is essential for successful plant establishment (Baskin and Baskin, 2014; Ludewig et al., 2014). Various environmental factors, especially temperature and soil moisture, influence seed germination, and consequently seedling establishment (Baskin and Baskin, 2014). Knowledge of seed germination responses to these two environmental factors is useful in predicting the response of a species to changes in its habitat and in formulating effective strategies for restoration (Fenner and Thompson, 2005).

Temperature affects the capacity for germination by regulating dormancy and the speed of germination in non-dormant seeds (Baskin and Baskin, 2014). Three cardinal temperatures, i.e., minimum/base (*T*_b_), optimum (*T*_o_), and maximum/ceiling (*T*_c_) generally have been used to describe the range of temperatures over which seeds of a particular species can germinate (Bewley et al., 2013). These cardinal temperatures for germination match germination timing to favorable conditions for subsequent seedling growth and development (Alvarado and Bradford, 2002), thus helping predict their current/future spatial distribution (Dürr et al., 2015). A review of the threshold values for germination of species worldwide concluded that species of tropical origin had a high T_b_ (crop species), while species originating from cool growing areas had a very low T_b_ (wild species, trees, and cool season legume crops) (Dürr et al., 2015). Trudgill et al. (2000) also found that tropical species had a higher T_b_ than temperate species. Seeds of Fabaceae species from the Qing-Tibetan Plateau, an area with low temperatures and relatively abundant rainfall, had a lower base and a lower optimal temperature for germination than those from the Alax Desert, an area with high summer temperatures and low amount of rainfall (Hu et al., 2015).

The effect of temperature on seed germination also can be modeled by thermal time (i.e., temperature accumulation above the base temperature for the completion of germination) model, which is a very efficient method for predicting timing of germination under fluctuating environmental conditions. This approach can be more useful for predicting plant development stages than calendar date when temperatures for germination or other plant development stages are outside the experimental data range (Slafer and Savin, 1991).

Soil moisture is another major factor determining when seeds germinate in the field (Baskin and Baskin, 2014). Bradford (2002) reported that germination occurs after non-dormant seeds have accumulated enough thermal time at a suitable water potential. The hydrotime model was formulated by Gummerson (1986) and Bradford (1990), and it mostly is used to analyze germination rates (speed) at different water potentials. Seed germination responses to water potential are species-specific (Fenner and Thompson, 2005; Baskin and Baskin, 2014), and different species and seed lots may have a different hydrotime constant (*θ*_H_) and base water potential (*ψ*_b_). Base water potential for 50% germination [*ψ*_b(50)_] is related to the ability of the embryo to overcome restraints to its growth imposed by the conditions of the embryo itself, the surrounding tissues, and the environment (Huarte, 2006). Seeds with low *ψ*_b(50)_ germinate better at low median water potential than those with high *ψ*_b(50)_. The base water potential at which seed germination can occur differs with species (Daws et al., 2008), and germination of species adapted to dry environments may be less affected by water stress than those adapted to wet environments (Allen et al., 2000). A study on seed germination of *Ammopiptanthus mongolicus*, *Glycyrrhiza uralensis*, *Lespedeza potaninii*, and *Sophora alopecuroides* from high summer temperatures and low amount of rainfall habitat and *Vicia amoena*, *V. angustifolia*, *V. sativa*, and *V. unijuga* from low temperatures and relatively abundant rainfall habitat suggested that the base median water potential differed among species but not between habitats (Hu et al., 2015).

*Stipa* species dominate large portions of the Eurasian zonal vegetation (Lavrenko and Karamysheva, 1993). Future projections of climate warming will impact the population dynamics of species by influencing seed germination and consequently their potential for natural regeneration (Markkj et al., 2009). Thus, research on how seed germination of *Stipa* species responds to different environments contributes to an understanding of where they grow, where they do not grow, and where they will be able to do so in the future (Dürr et al., 2015). Previous studies on *Stipa* species have usually focused on dispersal efficiency (Hensen and Müller, 1997), genetic diversity (Zhao et al., 2008; Jing et al., 2012), reproductive allocation (Tian et al., 2009), and cytology (Sheidai et al., 2006).

Seed germination response to temperature (Liu et al., 2011; Wang et al., 2011; Hu et al., 2012) and water potential (Huang et al., 2009; Zeng et al., 2010) of some species from the Qinghai-Tibet Plateau and the desert-grassland have been determined independently. However, comparison of germination responses of *Stipa* species from different habitats to temperature and substrate water potential have not been done. The purpose of our study was to compare responses to temperature and moisture of seeds of *Stipa* species from cool and warm habitats. We hypothesized that (1) seeds of *Stipa* species from habitats with low temperatures would have a lower *T*_b_, *T*_o_, and *T*_c_ than those from habitats with a relatively high temperatures, and (2) seeds of *Stipa* species from habitats with low amounts of rainfall are more tolerant of water stress than those from habitats with relatively high amounts of rainfall.

## Materials and Methods

### Seed Collection

Seeds of seven *Stipa* species were used in this study. Seeds of *Stipa grandis* were collected from Xilingol League in Inner Mongolia; *Stipa purpurea* from Guinan on the Qing-Tibetan Plateau; *Stipa penicillata* from Guoluo on the Qing-Tibetan Plateau; *Stipa breviflora*, *Stipa glareosa*, and *Stipa gobiea* from Alax in Inner Mongolia; and *Stipa bungeana* from Huanxian on the Loess Plateau. The species differ distinctly in habitat: *S. grandis*, *S. purpurea*, and *S. penicillata* occur in habitats with relatively abundant rainfall and relatively low temperature, whereas *S. bungeana*, *S. breviflora*, *S. glareosa*, and *S. gobiea* are important species in habitats with relatively low rainfall and relatively high temperature. See **Table 1** for additional information on the seven *Stipa* species.

**Table 1:** Detailed information on seeds of the seven *Stipa* species.

Habitat	Species	Seed collection time	Seed collection site	Mean annual temperature (°C)	Mean annual rainfall (mm)	1,000-seed weight (g)	Max. germination* (%)
cool	*Stipa grandis*	October	43°38′N 116°43′E, 1,400m	2.3	380	8.853	95.60 ± 0.27
	*Stipa purpurea*	October	35°43’N 100°49’E, 3,458m	2.3	404	2.353	97.17 ± 0.24
	*Stipa penicillate*	October	34°27’N 100°13’E, 3,730m	-4.0	443	1.425	97.56 ± 0.33
warm	*Stipa glareosa*	June	38°18’N 105°40’E, 1,560m	8.2	120	4.101	80.51 ± 0.33
	*Stipa breviflora*	June	38°55’N 105°39’E, 1,480m	8.2	120	1.511	95.64 ± 0.18
	*Stipa gobiea*	June	38°55’N 105°39’E, 1,480m	8.2	120	4.28	98.91 ± 0.25
	*Stipa bungeana*	June	37°07’N 106°49’E, 1,650m	9.2	300	1.045	93.28 ± 0.13

*maximum germination percentage after 6 months of dry storage.

Seeds of each of the seven species were collected from several hundred plants in 2016, and the awns were removed by hand in the laboratory. To avoid the potential effect of dormancy on germination results, seeds were allowed to after-ripen in a paper bag at room conditions (20%–45% relative humidity; 16–22°C) for 6 months before germination experiments were initiated.

### Effect of Temperature on Seed Germination

For each treatment and species, four replicates of 25 seeds were placed in 9-cm-diameter Petri dishes on two sheets of filter paper moisten with 5 ml of distilled water. Seeds were incubated in light (12 h each day under white fluorescent tubes with a mean photon irradiance at seed level of 60 µmol m^-2^ s^-1^, 400–700 nm) at 5, 10, 15, 20, 25, 30, 35, and 40°C. Germination (radicle protrusion) was monitored daily for 28 days.

### Effect of Water Potential on Seed Germination

Seeds were incubated at 20°C in light at water potentials of 0, −0.2, −0.4, and −0.6 MPa. Polyethylene glycol 6000 (PEG) solutions were prepared according to Michel and Kaufmann (1973), and water potential of the solutions was determined at 20°C using a Dew Point Microvolt meter HR-33T (Wescor, Logan, Utah, USA). For each treatment, four replicates of 25 seeds each were placed in 9-cm-diameter Petri dishes on two sheets of filter paper moistened with 5 ml of PEG solution or distilled water (control), and Petri dishes were sealed with parafilm to reduce the speed of evaporation of water. Seeds were transferred to new filter paper with fresh solution every 48 h to ensure relatively constant water potential. Germination (radicle protrusion) was monitored daily for 28 days.

### Data Analysis

The effects of temperature and of water potential on germination percentage and rate (1/t_50_) of each species were analyzed using GLM (General Linear Models) analysis based on binomial distribution using SPSS 22.0 software.

A linear model (Eqn. 1, 2) was used to estimate base temperature (*T*_b_), maximum or ceiling temperature (*T*_c_), and optimum temperature (*T*_o_) as described by Ellis et al. (1986) and Hu et al. (2015).

(Eqn. 1) $$1/t_{g}=(T-T_{b})/\theta_{1}$$

(Eqn. 2) $$1/t_{g}=(T_{c}-T)/\theta_{2}$$

For thermal time model construction at the suboptimal temperature range and at the supraoptimal temperature range, cumulative germination values [probit (g)] from each monitoring time and suboptimal/supraoptimal temperatures were pooled and regressed against a function of time (*t*_g_) and temperature (*T*) as per Eqn. 3 (Cheng and Bradford, 1999) and Eqn. 4 (Ellis et al., 1986), respectively (see Hu et al., 2015).

(Eqn. 3) $$\text{probit}\;(g)=[ln(T-T_{b})t_{g}-\ln(\theta_{T(50)})]/\sigma_{\theta T}$$

(Eqn. 4) $$\text{probit}\;(g)=[T+(\theta_{T}/t_{g})-T_{c(50)}]/\sigma_{Tc}$$

The hydrotime constant *θ*_H_ (MPa-days), actual seed water potential *ψ* (MPa), and base water potential *ψ*_b_(g) (MPa) were calculated using the hydrotime model (Gummerson, 1986; Bradford, 1990; Cheng and Bradford, 1999) as below:

(Eqn. 5) $$\theta_{H}=(\Psi -\Psi_{b(g)})t_{g}$$

(Eqn. 6) $$\text{probit}\;(g)=[\Psi -(\theta_{H}/t_{g})-\Psi_{b(50)}]/\sigma_{\Psi b}$$

## Results

### Effect of Temperature on Seed Germination

**Figure 1:**
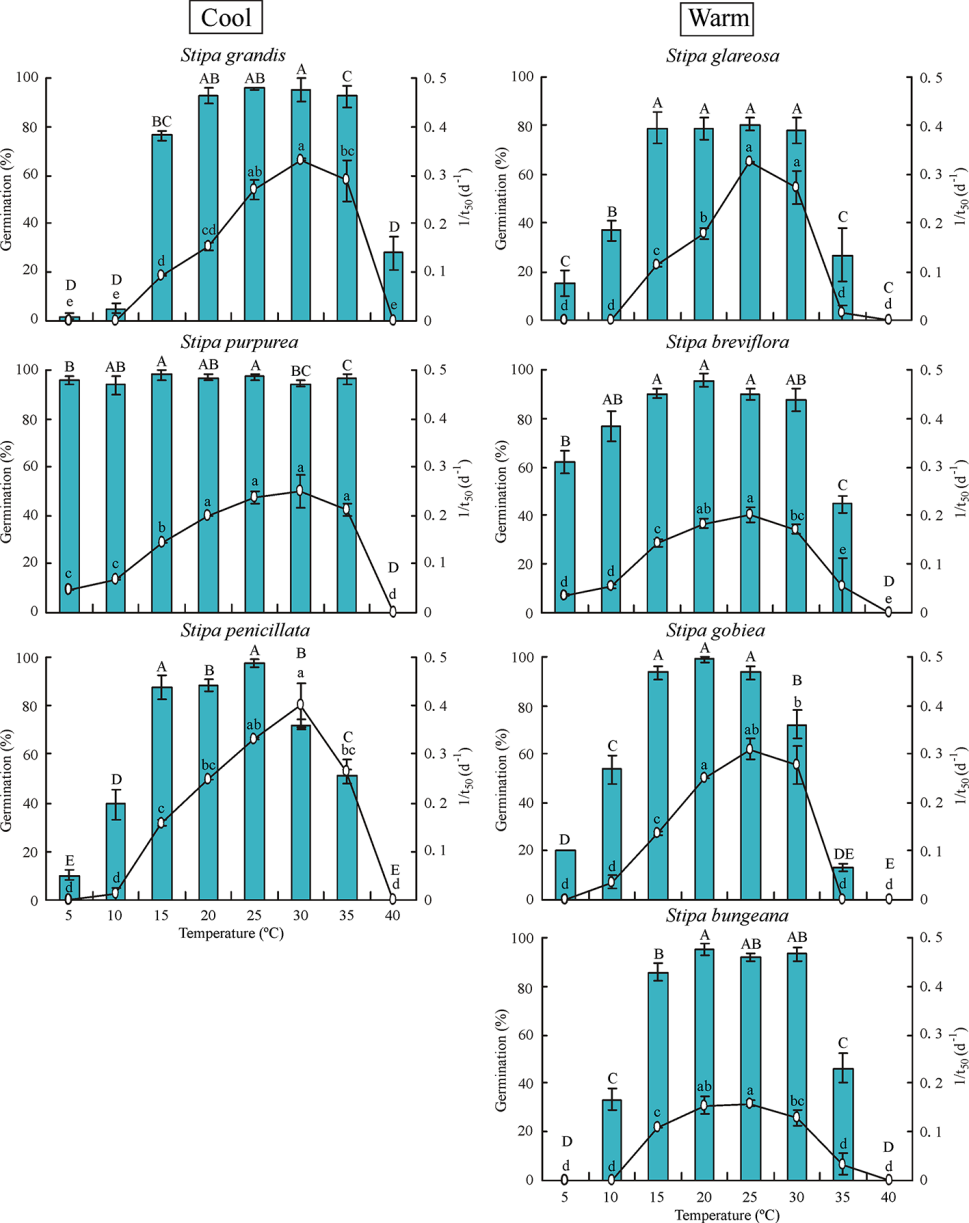
Seed germination percentage and rate (1/t_50_) of the seven *Stipa* species at eight temperatures. Bars with different uppercase letters differ significantly (*P* < 0.05) for germination percentage, and those with different lowercase letters differ significantly (*P* < 0.05) for germination rate. Cool species are on left and warm species on right, the same in **Figure 2** and **3**.

Temperature had significant effects (*P*< 0.05) on percentage and rate (1/t_50_) of germination of all seven *Stipa* species (**Figure 1**). In general, germination percentage increased and then decreased as temperature increased; however, *S. purpurea* seeds germinated to a high percentage (> 90%) at 5°C to 35°C. Germination of *Stipa* species from the cool habitats was more tolerant to high temperature than that of species from the warm habitats. For example, germination of *S. grandis*, *S. purpurea*, and *S. penicillata* seeds from habitats with low temperature and high rainfall (hereafter cool habitats) was 93%, 97%, and 51% at 35°C, respectively, whereas it was 27%, 45%, 13%, and 46% for seeds of *S. glareosa*, *S. breviflora*, *S. gobiea*, and *S. bungeana*, respectively, from habitats with high temperature and low amount of rainfall (hereafter warm habitats). However, germination of *Stipa* species at low temperature differed with species but not habitat. Seeds of *S. purpurea* from a cool habitat and those of *S. breviflora* from a warm habitat germinated to higher percentage at 5°C than *S. glareosa*, *S. gobiea*, and *S. penicillata*, while no seeds of *S. grandis* or *S. bungeana* germinated at 5°C. Seeds of *S. grandis*, *S. purpurea*, and *S. penicillata* from cool habitats had the highest germination rate at 30°C, whereas the highest rate for *S. bungeana*, *S. breviflora*, *S. glareosa*, and *S. gobiea* from warm habitats was at 25°C.

**Figure 2:**
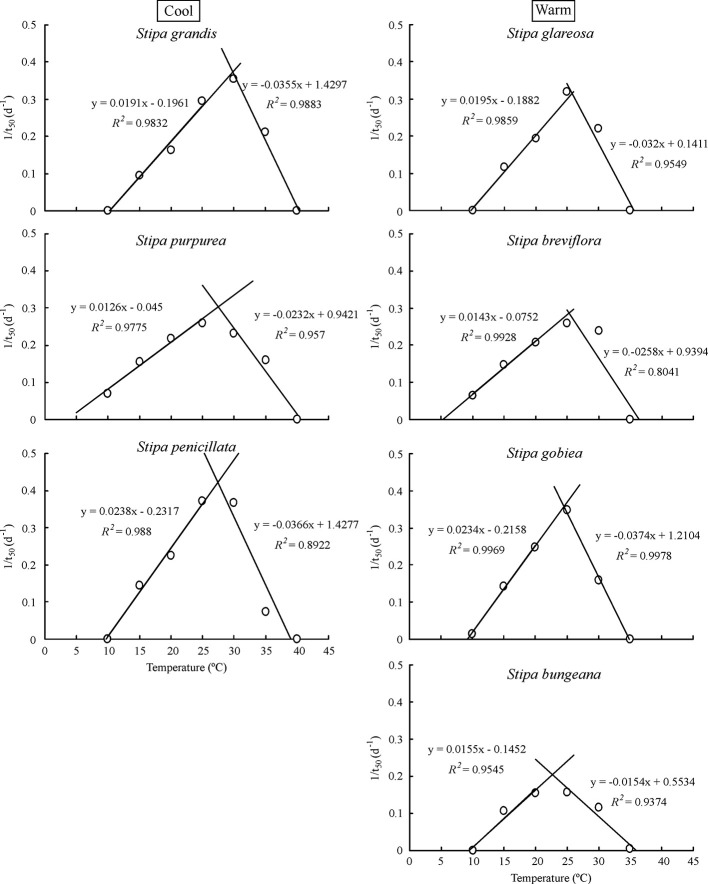
Linear regression of seed germination rate (1/t_50_) and temperatures at suboptimal and supraoptimal temperatures of the seven *Stipa* species.

Based on extrapolation, species from the cool habitats exhibited a higher *T*_o_ and *T*_c_ than those from warm habitats (**Figure 2**; **Table 2**). For example, *T*_c_ for *S. grandis*, *S. purpurea*, and *S. penicillata* from cool habitats was 40.8°C, 41.0°C, and 39.6°C, respectively, while *T*_c_ for *S. bungeana*, *S. breviflora*, *S. glareosa*, and *S. gobiea* from warm habitats was 36.9°C, 37.2°C, 35.7°C, and 35.6°C, respectively. Seeds of *S. breviflora* from a warm habitat and *S. purpurea* from a cool habitat had a lower *T*_b_ than the other species. *T*_b_ for *S. breviflora* and *S. purpurea* was 4.1°C and 3.8°C, respectively, while it was 10.3°C, 10.0°C, 9.9°C, 9.3°C and 9.7°C for *S. grandis*, *S. penicillata*, *S. glareosa*, *S. gobiea*, and *Stipa bungeana*, respectively.

**Table 2:** Estimation of the three cardinal temperatures for seed germination of the seven *Stipa* species using a linear regression of seed germination rate (1/t_50_) as a function of temperature.

Habitat	Species	*T*_b_	*T*_o_	*T*_c_
cool	*Stipa grandis*	10.3	30.1	40.8
	*Stipa purpurea*	3.8	28.2	41.0
	*Stipa penicillata*	10.0	28.1	39.6
warm	*Stipa glareosa*	9.9	26.1	35.7
	*Stipa breviflora*	4.1	25.1	37.2
	*Stipa gobiea*	9.3	25.0	35.6
	*Stipa bungeana*	9.7	23.3	36.9

T_b_ = base temperature, T_o_ = optimal temperature, T_c_ = maximum temperature.

Seed germination responses for all seven species were described well by the thermal-time model at suboptimal and supraoptimal temperatures. At suboptimal temperatures, thermal times (*θ*_T(50)_) for *S. grandis*, *S. purpurea*, *S. penicillata*, *S. glareosa*, *S. breviflora*, *S. gobiea*, and *S*. *bungeana* were 54.0 °C·d, 72.0 °C·d, 56.6 °C·d, 70.9 °C·d, 99.5 °C·d, 51.0 °C·d, and 84.5 °C·d, respectively. *θ*_T(50)_ values for these species at supraoptimal temperatures were 42.8 °C·d, 57.6 °C·d, 15.5 °C·d, 11.1 °C·d, 35.1 °C·d, 9.2 °C·d, and 68.0 °C·d, respectively. Consistent with estimation from extrapolation, species from cool habitats had a higher *T*_c_ than those from warm habitats. However, there was no difference between species from the two habitats in *T*_b_, *θ*_T(50)_, and *T*_c_ (**Tables 2**–**4**).

**Table 3:** Seed germination parameters of the seven *Stipa* species from cool and warm habitats based on thermal-time model analysis at suboptimal temperature.

Habitat	Species	*θ*_T(50)_(°C·d)	*σ*_θT_	*T*_b_ (°C)	*R* ^2^
cool	*Stipa grandis*	54.0	0.92	11	0.62
	*Stipa purpurea*	72.0	0.79	3	0.65
	*Stipa penicillata*	56.6	0.86	8	0.86
warm	*Stipa glareosa*	70.9	0.40	8	0.82
	*Stipa breviflora*	99.5	0.68	5	0.80
	*Stipa gobiea*	51.0	0.54	8	0.91
	*Stipa bungeana*	84.5	0.65	8	0.89

θ_T(50)_ = thermal time for 50% of seeds to germinate, σ_θT_ = standard deviation for θ_T(50)_, T_b_ = constant base temperature in suboptimal temperature range.

**Table 4:** Seed germination parameters of the seven *Stipa* species from cool and warm habitats based on thermal-time model analysis at supraoptimal temperature.

Habitat	Species	*θ*_T_(°C·d)	*σ*_θT_	*T*_c(50)_(°C)	*R* ^2^
cool	*Stipa grandis*	42.8	7.40	37	0.83
	*Stipa purpurea*	57.6	10.10	44	0.99
	*Stipa penicillata*	15.5	10.60	35	0.98
warm	*Stipa glareosa*	11.1	2.50	32	0.91
	*Stipa breviflora*	35.1	6.00	36	0.97
	*Stipa gobiea*	9.2	3.90	31	0.99
	*Stipa bungeana*	68.0	7.40	37	0.93

θ_T_ = constant thermal time, σ_θT_ = standard deviation for T_c(50)_ at supraoptimal temperature, T_c_ = maximum temperature for 50% of seeds to germinate.

### Effect of Water Potential on Germination

**Figure 3:**
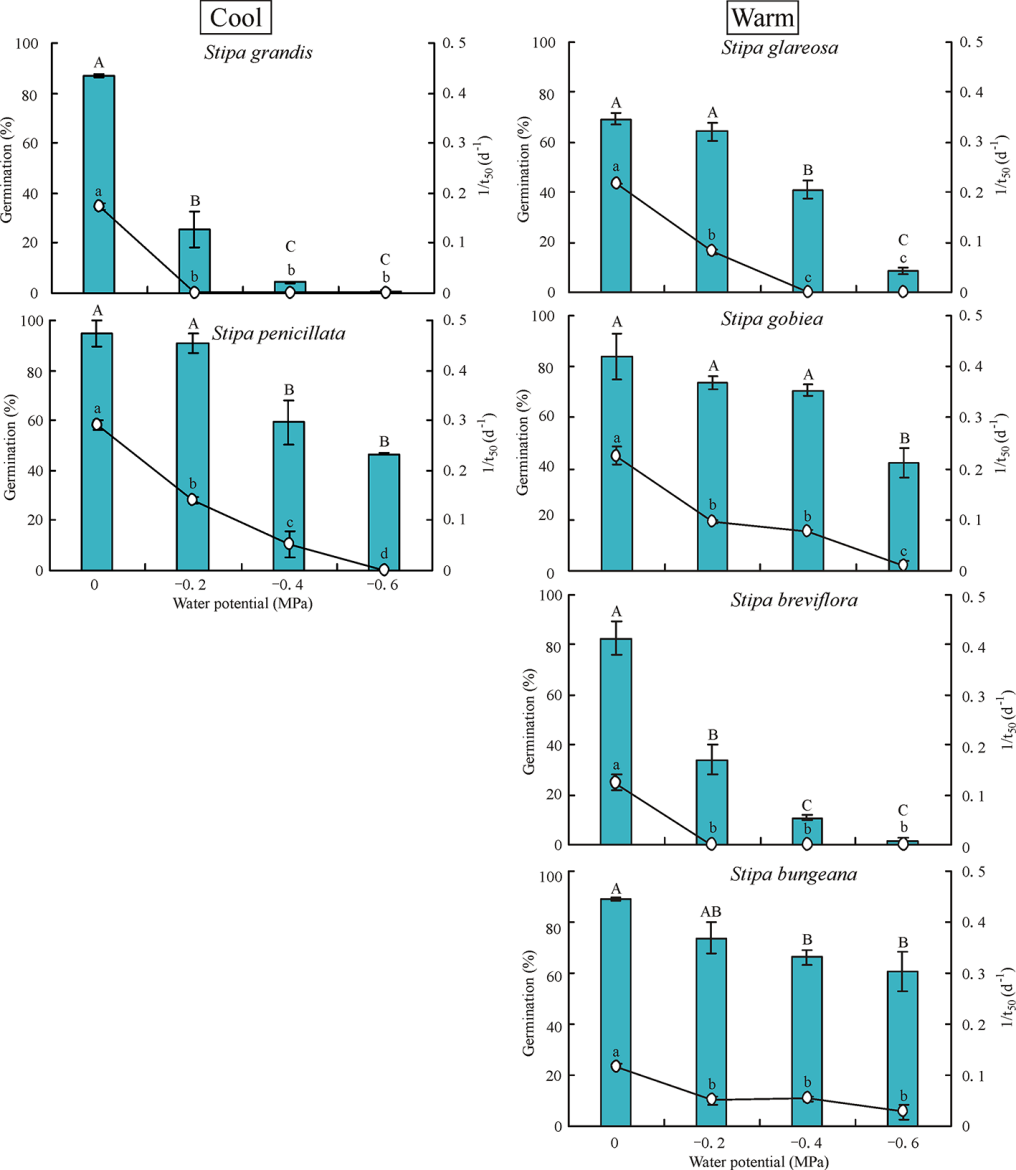
Seed germination percentage and rate (1/t_50_) of six *Stipa* species tested at four water potentials. Bars with different uppercase differ significantly (*P* < 0.05) for germination percentage, and those with different lowercase letters differ significantly (*P* < 0.05) for germination rate.

Water potential had a significant effect on germination percentage and rate of the six *Stipa* species tested (**Figure 3**). Germination percentage decreased significantly as water potential decreased. Seeds of *S. grandis* and *S. breviflora* did not germinate at -0.6 MPa, and those of *S. glareosa* germinated to only 8.7%. However, seeds of *S. gobiea*, *S. bungeana*, and *S. penicillate* germinated to 42%, 61%, and 47%, respectively, at -0.6 MPa.

The hydrotime models described germination of the *Stipa* species well (**Table 5**). *S. bungeana* had the lowest *ψ*_b(50)_ (-0.73 MPa) and *S. grandis* the highest *ψ*_b(50)_ (-0.21 MPa). The hydrotime constant (*θ*_H_) differed with species. Species with low *ψ*_b(50)_ exhibited a high *θ*_H_, i.e., 173 MPa·d for *S. bungeana* and 85 MPa·d for *S. gobiea*. However, *S. grandis* with a high *ψ*_b(50)_ exhibited a low *θ*_H_, i.e., 31 MPa·d. There was a negative correlation between *ψ*_b(50)_ and *θ*_H_ across species tested. 

**Table 5:** Seed germination parameters for response of the six tested species of *Stipa* from cool and warm habitats to water potential based on hydrotime model analysis.

Habitat	Species	*θ*_H_ (MPa·d)	*ψ*_b(50)_	*σ*_ψb_	*R*²
cool	*Stipa grandis*	31	-0.21	0.12	0.94
	*Stipa penicillata*	62	-0.62	0.21	0.89
warm	*Stipa glareosa*	56	-0.41	0.17	0.93
	*Stipa breviflora*	36	-0.19	0.14	0.91
	*Stipa gobiea*	85	-0.61	0.22	0.83
	*Stipa bungeana*	173	-0.73	0.28	0.83

θ_H_ = constant hydrotime, ψ_b(50)_ = base water potential for 50% of seeds to germinate, σ_ψb_ = standard deviation of ψ_b(50)_.

## Discussion

We showed that germination of seeds of the *Stipa* species from cool habitats had a higher base (*T*_b_), optimal (*T*_o_), and maximum (*T*_c_) temperature than species from warm habitats, which is contrary to our first hypothesis. Further, the germination responses to water potential differed among the six species tested but not between habitats, which does not agree with our second hypothesis. There was a negative correlation between hydrotime constant (*θ*_H_) and base water potential for 50% of the seeds of all the species to germinate (*ψ*_b(50)_).

### Germination Responses to Temperature of *Stipa* Species From Different Habitats

Major differences in germination traits depend on climate conditions where the species grow or originated, with pea seeds being able to germinate on ice (Stupnikova et al., 2006) and some crop species of tropical origin unable to germinate below 18°C (Dürr et al., 2015). The germination responses of seeds to temperature may differ among species (Allen et al., 2000; Daws et al., 2010) and climate conditions where the species grows (Cochrane et al., 2014). Tropical species such as *Echinochloa crusgalli*, *Panicum miliaceum*, and some perennial warm-season forage grasses (Hsu and Nelson, 1986) usually require a higher base temperature for germination than species such as *Lotus corniculatus* (Hur and Nelson, 1984), *Hordeum vulgare*, and *Avena sativa* (Trudgill et al., 2000) that originate from temperate regions. Further, species such as *Vicia sativa* and *Senecio diversipinnus* from cool-habitat growing conditions have a lower base temperature than species such as *Sophora alopecuroides* and *Senecio squalidus* from temperate regimes (Liu et al., 2011; Wang et al., 2011; Hu et al., 2015). A review of seed germination data for 243 species (crops, horticulture, range and forages, trees) showed that the highest base temperatures were for crop species of tropical origin, such as cotton, pearl millet, and mungbean and that the lowest values often were for wild species/trees originating from cool growing-season areas (Dürr et al., 2015).

In contrast, we found that seeds of *Stipa* species from cool habitats had a higher base temperature than those from warm habitats, except for *S. purpurea*. Billings and Mooney (1986) reported that germination temperatures for alpine species are high, which may prevent germination too early or too late for seedling establishment in the brief summer. Seedling death is reduced after snowmelt when germination is triggered by relatively high temperature in late spring or early summer (Alvarado and Bradford, 2002; Shimono and Kudo, 2005). This result agrees with the study by Rosbakh et al. (2015) that the base temperature is strongly negatively correlated with habitat temperature. However, for *S. purpurea*, a species that occurs in alpine grasslands on the Qing-Tibetan Plateau, the base temperature was 3.8°C, which was lower than that of *S. grandis* (10.3°C) and *S. penicillata* (10.0°C) from the same cool habitat. Morever, seeds of *S. purpurea* collected in 2013 from alpine steppe on the Qing-Tibetan Plateau had a base temperature 0.1°C lower than that of this species in the present study (Yang et al., 2014). It previously has been shown that species with a wide geographical distribution often exhibit a large variation in germination characteristics among seed provenances (Keller and Kollmann, 1999; Fenner and Thompson, 2005) and seed collection years (Andersson and Milberg, 1998; Beckstead et al., 2011). The difference in *S. purpurea* may be due to seeds of this species being collected from different populations/years, and this issue requires further study.

The optimal and maximum temperatures for germination of *Stipa* species from cool habitats were higher than those of species from warm habitats. Seeds of *S. grandis*, *S. purpurea*, and *S. penicillata* from cool habitats germinated best at high temperatures, indicating that when seeds matured in autumn temperatures are too low for germination, which would prevent seedlings from emerging and possibly being killed by freezing in winter (Wesche et al., 2006). Genetics and the environment of the mother plant during seed development are two important factors controlling variation in germination requirements within populations of a species (Baskin and Baskin, 2014). Different *Stipa* species growing in different geographical regions and their distribution is positively correlated with temperature (Yang et al., 2014)*. S. purpurea* is the dominant species in alpine steppe on the Tibetan Plateau. Xiong et al. (2014) reported that the optimum temperature regime for germination of this species ranged from 15°C to 25°C and that temperatures higher than 30°C would not to be conducive to seed germination. However, in our study the optimum temperature for germination of *S. purpurea* was 30°C. Two possible reasons for the difference in optimum temperature are that environmental conditions of the two seed collection sites differed and/or there is genetic differentiation in germination between the two populations (Baskin and Baskin, 2014). Consistent with the present study, Ronnenberg et al. (2007) reported that seeds of five mountain steppe species (but not of *Stipa*) from central Asia germinated best at high temperature (20/32°C).

### Germination Responses to Water Potential of *Stipa* Species From Different Habitats

The ability to germinate at low water potential often is interpreted as an adaptation to dry habitats (Tobe et al., 2001; Bochet et al., 2007). However, the relationship between the germination responses of a seed and water stress/tolerance of the plant cannot be generalized (Daws et al., 2008; Zeng et al., 2010). For instance, seeds of *Caragana korshinskii* (15.0% germination at 2.1 MPa) and *Hedysarum scoparium* (6.5% germination at 1.8 MPa) from semi-arid regions of northwest China, and *Reaumuria soongorica* (2.2% germination at 1.8 MPa) from arid regions have a higher germination capacity at lower water potential than seeds of *Artemisia sphaerocephala* (8.3% germination at 1.5 MPa) from semi-arid regions and *Zygophyllum xanthoxylum* (5.2% germination at 1.2 MPa) from arid regions. However, seedling establishment of *C. korshinskii*, *H. scoparium*, and *R. soongorica* is lower in arid regions than that of *A. sphaerocephala* and of *Z. xanthoxylum* (Zeng et al., 2010).

Generally, species from dry habitats are more tolerant of water stress than those from wet habitat (Evans and Etherington, 1990; Allen et al., 2000). Ludewig et al. (2014) concluded that selection pressure from soil moisture stress might be lower for species from wet habitats than for those from dry habitats. Thus, under experimental conditions species from wet habitats can germinate under dry conditions. *ψ*_b(50)_ of the six *Stipa* species tested was species-specific but was not habitat-specific.

Species from warm habitats differ in germination response to water potential, and the rank-order of tolerance to water stress was *S. bungeana* (-0.73MPa) > *S. gobiea* (-0.61MPa) > *S. breviflora* (-0.19 MPa). Thus, there was no clear pattern in response to water potential in relation to habitat type, which is consistent with the results obtained by Daws et al. (2008) for germination of Neotropical species. The possible reason for within habitat (warm) species differences in germination of *Stipa* in our study may be that soil moisture during germination is similar between the warm and cool habitats, although the annual mean rainfall differed greatly between them (Hu et al., 2015). Moreover, Allen et al. (2000) reported that seeds with a low *θ*_H_ and high *ψ*_b(50)_ may have a rapid germination rate with no water stress but are strongly inhibited at low water potentials. For example, seeds of *S. grandis* and *S. breviflora* have high *ψ*_b(50)_ and low *θ*_H_. In our study, there was a negative correlation between *θ*_H_ and *ψ*_b(50)_, indicating that seeds with high tolerance to water stress (low *ψ*_b(50)_) need more time to germinate than those with high *ψ*_b(50)_. A high *θ*_H_ for germination may play an important role in preventing seed germination after a low precipitation event that is followed by drought, which would kill the seedlings (Hu et al., 2015).

## Conclusions

The thermal time and hydrotime models performed well in predicting seed germination time for non-dormant (after-ripened) seeds of the seven *Stipa* species studied. Temperature requirements for germination, but not for water potential, of the *Stipa* species were strongly related to habitat type. Seeds of *Stipa* species from cool-wet habitats were more tolerant of high temperatures than those from warm-dry habitats. These results help us to better understand the germination requirements of these species and provide useful information for grassland restoration. However, our study included only seven *Stipa* species and two habitat types. Thus, information is needed for more species, with careful attention being given to habitat macroclimate.

## Data Availability Statement

All datasets presented in this study are included in the article/supplementary material.

## Author Contributions

XH and YW conceived the topic. RZ and KL performed the experiments. RZ and DC analyzed all statistical data. XH and RZ wrote the manuscript. JB and CB revised the manuscript. All authors contributed to the article and approved the submitted version.

## Conflict of Interest

The authors declare that the research was conducted in the absence of any commercial or financial relationships that could be construed as a potential conflict of interest.
